# UR-Net: An Integrated ResUNet and Attention Based Image Enhancement and Classification Network for Stain-Free White Blood Cells

**DOI:** 10.3390/s23177605

**Published:** 2023-09-01

**Authors:** Sikai Zheng, Xiwei Huang, Jin Chen, Zefei Lyu, Jingwen Zheng, Jiye Huang, Haijun Gao, Shan Liu, Lingling Sun

**Affiliations:** 1 Ministry of Education Key Laboratory of RF Circuits and Systems, Hangzhou Dianzi University, Hangzhou 310018, China; zhengsk@hdu.edu.cn (S.Z.); chenjin272@hdu.edu.cn (J.C.); lvzefei@hdu.edu.cn (Z.L.); jingwen@hdu.edu.cn (J.Z.); hjynet@hdu.edu.cn (J.H.); sunll@hdu.edu.cn (L.S.); 2 Sichuan Provincial Key Laboratory for Human Disease Gene Study, Sichuan Academy of Medical Sciences & Sichuan Provincial People’s Hospital, University of Electronic Science and Technology of China, Chengdu 610072, China; shanliusyy@uestc.edu.cn

**Keywords:** WBCs classification, stain-free, image enhancement, convolutional neural network, UR-Net, attention mechanism

## Abstract

The differential count of white blood cells (WBCs) can effectively provide disease information for patients. Existing stained microscopic WBC classification usually requires complex sample-preparation steps, and is easily affected by external conditions such as illumination. In contrast, the inconspicuous nuclei of stain-free WBCs also bring great challenges to WBC classification. As such, image enhancement, as one of the preprocessing methods of image classification, is essential in improving the image qualities of stain-free WBCs. However, traditional or existing convolutional neural network (CNN)-based image enhancement techniques are typically designed as standalone modules aimed at improving the perceptual quality of humans, without considering their impact on advanced computer vision tasks of classification. Therefore, this work proposes a novel model, UR-Net, which consists of an image enhancement network framed by ResUNet with an attention mechanism and a ResNet classification network. The enhancement model is integrated into the classification model for joint training to improve the classification performance for stain-free WBCs. The experimental results demonstrate that compared to the models without image enhancement and previous enhancement and classification models, our proposed model achieved a best classification performance of 83.34% on our stain-free WBC dataset.

## 1. Introduction

Blood detection plays a significant role in the diagnosis and treatment of diseases. As an important part of blood, white blood cells (WBCs) can resist bacteria and viruses in the human body and are also referred to as immune cells [1]. According to their morphological structure, WBCs can usually be roughly classified into three types: granulocytes, monocytes, and lymphocytes [2]. The content of WBCs in blood is closely related to various blood diseases, which can be used as a standard for diagnosing the category and severity of diseases, such as leukemia [3] and cancer [4]. Therefore, research on the classification and counting of WBCs is of great value for medical diagnosis [5].

Previous WBC classification was generally achieved by professional medical personnel using blood smears [6], and its accuracy greatly depended on the medical personnel’s knowledge and experience. In recent years, the widespread application of deep learning (DL) has enabled computers to assist humans better in completing complex tasks. The classification of WBCs through convolutional neural networks (CNNs) not only reduces the workload of professionals but also has higher accuracy than humans [7]. Nevertheless, most of the current deep learning-based WBC classification is applied to stained WBCs, since the stained cells have clearer contrast and nuclear features under the microscope [8] while stain-free cells are not conducive to CNN for classification. However, the stained WBCs classification has the following obvious disadvantages: (1) The preparation of reagents required for staining takes a long time; (2) the staining process may cause irreversible effects on cells so that their morphological features will be different from the original ones; (3) this operation still requires professionals. Because of these shortcomings, stain-free WBC classification has become a research hotspot in the field of bioimaging [9,10].

For the acquisition of stain-free WBC images, microscopy is still the most convenient instrument for obtaining blood smear images. However, due to the influence of external factors such as illumination [11], the images obtained directly by the microscope have the issue of low quality, which seriously affects the classification performance of the subsequent neural network. Therefore, as one of the preprocessing methods for image classification, image enhancement is essential to improving image quality. In recent years, image enhancement techniques have been largely investigated, especially in the field of medical imaging. Many traditional approaches use histogram equalization [12,13], sharpening filtering [14], super-resolution [15,16], Retinex [17], etc., to enhance the image, while others apply homomorphic filtering [18], and wavelet transform [19,20] to process the image in the frequency domain. Several more recent works have shown that CNN can successfully demonstrate better performance and efficiency compared with traditional methods in image enhancement [21,22]. The above methods have indeed achieved effective results in improving human visual sense, but they do not necessarily perform well in computer vision tasks such as classification or object recognition. Thus, the latest research is to integrate CNN image enhancement techniques into the neural network, the purpose of which is to improve the classification performance, rather than human perception [23].

In this paper, we first conducted experiments on our WBC dataset by pairing each of the UNet, UNet++, ResUNet as the enhancement network with VGG16, MobileNetV2, Dense-Net121 and ResNet101 as the classification network, respectively. Among them, the combination of ResUNet and ResNet101 has the best performance. Therefore, we propose a novel network architecture, UR-Net, which jointly employs ResUNet as the framework for image enhancement and ResNet101 for classification. The proposed network integrates the enhancement model with the classification model, allowing joint training to enhance both the image quality and the classification performance for stain-free WBCs. The ResUNet structure through downsampling and upsampling to generate new images. On this basis, we replaced certain layers to enhance network stability during training. Then, we added a convolutional layer in the cross-layer connection between downsampling and upsampling to optimize the fusion of shallow and deep features. Moreover, we incorporated attention mechanisms [24] in the upsampling process to emphasize the features of WBCs while mitigating the impact of background noise on network classification performance. Finally, different from previous image enhancement networks that do not require pre-training methods due to their simple network structures, we have employed pre-training for the enhanced ResUNet, which exhibits a more intricate structure in our joint network. Specifically, we pre-trained the modified ResUNet by setting the input and output images as the same image to obtain a better initial weight value to expedite convergence. As a result, we achieved an optimal accuracy of 83.34%.

The main contributions of the present study can be summarized as follows:

(1) A novel network architecture, UR-Net, was proposed for stain-free WBC image classification, which jointly employs ResUNet as the framework for image enhancement and ResNet101 for classification.

(2) The purpose of the proposed image enhancement technology is to improve WBC classification performance, rather than human visual perception.

(3) The pre-training approach employed for image enhancement networks facilitates faster convergence and achieves higher accuracy within a limited number of training epochs.

(4) The proposed method achieved a higher accuracy compared to previous studies in the existing literature.

The remainder of this paper is organized as follows. Section 2 provides a brief review of the related works. Section 3 describes our proposed enhancement architecture and training procedure. In Section 4, the experimental results are presented and discussed through various comparative experiments. The article is concluded in the last Section 5.

## 2. Related Works

### 2.1. Traditional Image Enhancement

Traditional image enhancement can be classified into two categories according to different implementation methods: spatial domain enhancement and frequency domain enhancement. The spatial information of the image can reflect the position, shape, and size of the objects in the image. Shahzad et al. [25] utilized adaptive histogram equalization to improve the contrast of WBC images in preprocessing, then classified WBCs via a CNN with an ant colony algorithm. The sharpening filter can attenuate low-frequency components in the image to enhance the edge information of the image. Pham et al. [26] introduced a method that integrated anisotropic averaging with the Laplacian kernels for grayscale image sharpening to determine the optimal interpolation weights in the spatial domain. The opposite of a sharpening filter is a smoothing filtering, which can attenuate high-frequency components in the image, so it can be applied to image denoising. Li et al. [27] proposed a smoothing filtering with a weighted guided image filter, improving artifacts while denoising. Super-resolution (SR), as another technology to improve image quality, is the transformation of an image from low resolution (LR) to high resolution (HR). Traditional SR techniques usually use interpolation [28] to improve image quality. Other methods, such as Retinex [29], can mitigate the effect of the light source, thereby improving the quality of the image.

The frequency domain enhancement requires transforming the image from the spatial domain to the frequency domain. Homomorphic filtering can remove multiplicative noise and increase contrast. Khan et al. [30] indicated that adaptive Homomorphic filters can work well for ultrasound images degraded with higher values of speckle noise. A widespread approach, wavelet transform, can divide the image signal into different frequency bands and enhance the signals in different frequency bands at the same time. Cao et al. [31] modified the discrete wavelet transform and proposed an enhanced three-dimensional discrete wavelet transform approach to extract the feature, alleviate the noise, and adopt a CNN model for classification subsequently.

### 2.2. CNN-Based Image Enhancement to Improve Human Perception

In contrast to traditional enhancement algorithms, DL techniques can successfully simulate extensive image enhancement by training on pairs of input and target output images. The target output images are usually acquired by state-of-the-art instruments, while the input images are acquired by some low-precision instruments. The strategy is to train the input image via CNN to approximate the output of the corresponding target image. Huang et al. [32] presented a novel UNet structure, the range scaling global UNet (RSGUNet), for images from mobile devices to improve human perception. Meanwhile, they used the digital single-lens reflex (DSLR) camera to acquire the target image corresponding to the low-quality images. Similar to this work, Ignatov’s group [33] used a residual CNN to improve both color rendition and image sharpness. However, there are other approaches that are only used in specific situations. Lore et al. [34] proposed a deep network, which is one of the first DL approaches to enhance low-light images (LLI). Its architecture was based on a deep autoencoder to identify signal features from LLI and adaptively brighten images. Su et al. [35] proposed a residual network via multi-scale cross-path concatenation to suppress the noise. Chakrabarty et al. [21] presented a new method using a neural network trained for blind motion deblurring.

SR technology also achieves excellent performance on CNN. Existing SR methods were often focused on network selection. Reshad et al. [36] via a generative adversarial network (GAN) to generate a sufficient dataset, then used a CNN to learn an end-to-end mapping from LR to HR. The purpose was to enhance the sensing images. Huang et al. [37] propose a single-image super-resolution neural network that exploits the mixed multi-scale features of the image, which can extract local texture features and global structural features and achieves higher performance with fewer parameters.

### 2.3. CNN-Based Image Enhancement to Improve Neural Network Classification Performance

Neural networks are derived from neuroscience and cognitive science, but they have many differences in the process of dealing with problems. Therefore, even though the above methods can greatly improve the observer’s perceived quality of images, they may not necessarily improve the performance of computer vision tasks. To understand how neural networks process images, Dodge and Karam [38] analyzed how blur, noise, contrast, and compression hinder the performance of CNN. Their experiments showed that CNN was very sensitive to blur and noise, but resilient to compression distortions and contrast changes. Ullman et al. [39] compared how well humans and CNNs recognize minimal recognizable images, demonstrating that a minute change in the image can have a drastic effect on computational recognition.

To improve the performance of CNN, Sharma et al. [23] first propose a unified CNN architecture that uses a range of enhancement filters that can enhance image-specific details via end-to-end dynamic filter learning. Their overall goal is to improve image classification rather than human perception. To solve the low-light problem, Al Sobbahi et al. [40] integrated the homomorphic filter into CNN and obtained the best filter parameters through the learning of the network.

A major issue is that the application of this method is still based on one or more filters. Although the parameters of the filter can be learned well via CNN to achieve the best accuracy, the final effect is still based on the function of the filter itself. Moreover, most of the existing methods are applied to natural images, not medical images.

## 3. Methods

### 3.1. Dataset

**Figure 1:**
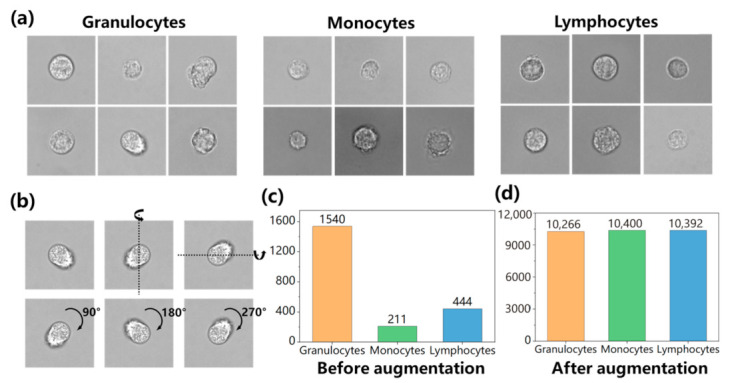
Dataset processing. (**a**) Part of three-type WBC images in the datasets after segmentation. (**b**) Process of dataset enhancement. The first row is the raw image, horizontally and vertically flipped image. The second row is the image rotated clockwise at different angles. (**c**) Datasets before augmentation. (**d**) Datasets after augmentation.

Our raw stain-free WBC image data were obtained from previous work [10], where the blood samples were collected from multiple healthy donors. These blood donors reported general health and no use of medical prescriptions in the last 2 weeks before enrollment. We separated the blood samples into red blood cells and WBCs using a microfluidic chip based on a spiral channel. The separated WBCs were subsequently fluorescently stained, and both brightfield and fluorescence images were acquired using a 100× objective lens within the same field of view. The fluorescence imaging allowed for the visualization of details pertaining to the nuclei of WBCs, which was used to differentiate between the actual types of each corresponding bright-field image of WBCs. Finally, the brightfield images were segmented into 200 × 200 sizes to form the training or testing dataset. A more detailed description of the dataset collection process can be referred to in [10]. Part of the dataset after segmentation is shown in Figure 1a.

As shown in Figure 1c, there are significant differences in the number of data belonging to different categories, which is due to the inherently unbalanced numbers of WBCs in human blood. To ensure the independence of the training and testing sets, we segregated the original WBC dataset into training and testing subsets at an 8:2 ratio. After that, we implemented rotation and flipping techniques to augment approximately 10,000 images of the three types of cells, as shown in Figure 1b, to mitigate overfitting concerns that may arise from small datasets and bias concerns that may arise from data imbalance. The number of augmented datasets is shown in Figure 1d.

### 3.2. UR-Net

**Figure 2:**
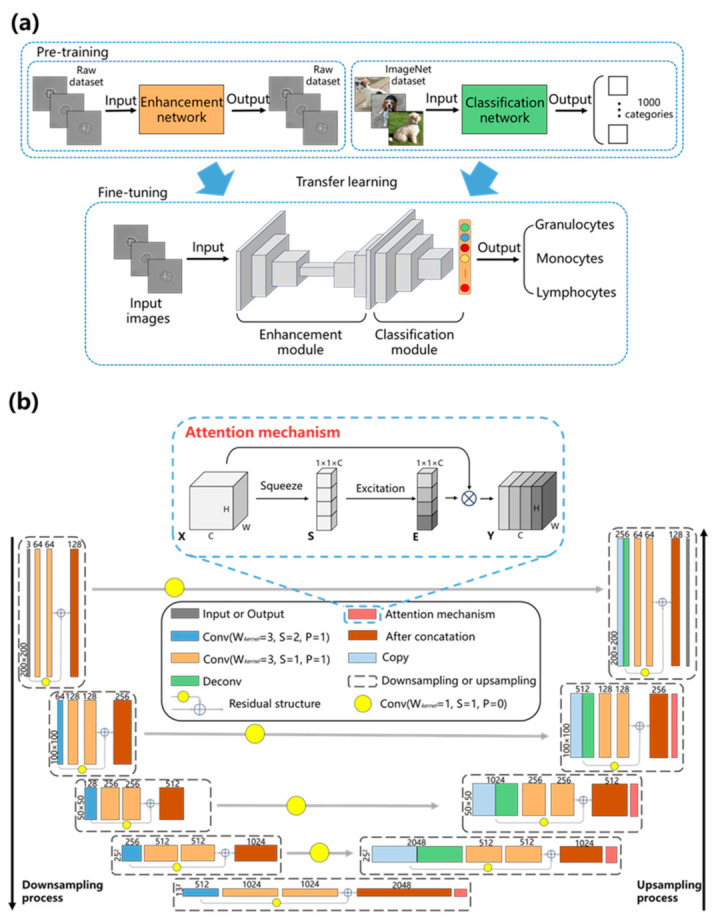
UR-Net. (**a**) Workflow of the proposed UR-Net model. (**b**) Architecture of modified enhancement module.

The UR-Net model proposed in this paper consists of two modules, as shown in Figure 2a. The first module is an image enhancement network constructed by the ResUNet framework, and the second module employs ResNet101 as the classification network. The image enhancement network is seamlessly embedded in the ResNet classification network, making it an end-to-end stain-free WBC classification network. ResUNet is an evolution of UNet that incorporates residual structures from ResNet. Its architecture can be seen in Figure 2b. This module comprises downsampling and upsampling processes. In the downsampling process, each downsample involves a pooling layer and two convolutional layers that extract different features of WBC images. By stacking consecutive convolutional layers, the network can focus more on global information in the images. After four downsamples, the upsampling process restores the image to its original size through each upsampling, which includes a deconvolutional layer and two convolutional layers. This provides sufficient learning space for the neural network to enhance its own features. During the upsampling process, cross-layer connections are used to fuse shallow and deep features to compensate for any loss of edge information caused by downsampling. Finally, residual structures are applied to the upsampling and downsampling processes to enhance feature transmission for WBCs.

In order to focus ResUNet more on image feature enhancement, we modified ResUNet, as shown in Figure 2b. First, due to the convolution operation, the output feature map may differ in size from the input. To facilitate the residual connections of the structure, as well as inter-layer connections during the upsampling and downsampling processes, we select a convolution kernel parameter of 3 with padding of 1 and stride of 1 to alleviate memory consumption on the computer.

Second, the pooling layer is replaced by the convolutional layer. Although the pooling layer can enhance the robustness of the model and prevent overfitting during training, it also discards some features. For stain-free WBC images, which have fewer inherent features compared with fluorescently stained WBC images, some useful features may be discarded by the pooling layer, thereby limiting the feature extraction capabilities of the network. Therefore, we modified the pooling layer to a convolutional layer and adjusted its parameters to reduce the feature map to half of its input size.

Third, LeakyReLU is employed as the activation function. The ReLU function has performed well in many tasks, but its neurons are more likely to become inactive during training. LeakyReLU maintains a small gradient when x < 0, avoiding the problem of neurons not being activated.

Furthermore, in the cross-layer connections between down-sampling and up-sampling, we introduced a 1 × 1 convolutional layer. Although simple copying of feature maps for connection can effectively preserve shallow features, especially edge information, such rough connections may lead to ineffective mixing with deep feature maps. Therefore, the applied 1 × 1 convolutional layer can provide a buffering stage for this fusion process and facilitate better learning of shallow features during backpropagation.

Finally, we added a channel attention mechanism [41] before each deconvolutional layer. The channel attention mechanism is used to control and adjust the importance of feature representations for each channel, adaptively highlighting important features in different channels through learning dynamic weights, in order to better capture key information in WBC images or feature maps. The lighting conditions of microscopy greatly affect the background of the acquired WBC dataset images, posing significant challenges to the effective learning of the network. This attention mechanism enables the model to enhance useful features and suppress useless ones, thereby directing the focus of the model on the cells themselves rather than the background.

As shown at the top of Figure 2b, the channel attention mechanism consists of two operations: squeeze and excitation. The squeeze operation encodes the entire spatial feature of a channel on the input feature map as a global feature. We set the input feature vector as *X*, $X=\left[x_{1},x_{2},\ldots ,x_{c}\right],\;\;X\in R^{H\times W\times C}$, and the output of squeeze operation *S*, $S=\left[s_{1},s_{2},\ldots ,s_{c}\right],\;\;S\in R^{C}$. The formula is shown in (1),

(1) $$s_{c}=f_{squeeze}(x_{c})=\frac{1}{H\times W}\sum_{i=1}^{H}\sum_{j=1}^{W}x_{c}(i,j),$$

where *H* and *W* represent the height and width of the feature map, respectively. Then, excitation operations are applied to the feature vectors obtained in the previous step through two fully connected layers, $L_{1}$ and $L_{2}$, to learn weights that amplify or attenuate each channel, thereby extracting salient features from the channels. In the attention mechanism of Figure 2b, grays of different depths correspond to different weights after amplification or attenuation. The excitation formula is shown in (2),

(2) $$E=f_{excitation}(Z,L)=\sigma (L_{2}\delta (L_{1}Z)),$$

where $E=\left[e_{1},e_{2},\ldots ,e_{c}\right],\;\;E\in R^{C}$ is the output of the excitation operation. *L* represents two full connection layers, σ and δ represent the sigmoid and ReLU activation functions respectively. In the end, the output of channel attention mechanism *Y* is obtained by multiplying *E* and *X*.

For classification networks, ResNet101 has achieved high ratings on many classification tasks, due to its residual architecture. So, in this work, we transferred ResNet101 whose parameters were learned well on the ImageNet dataset [42]. Then, we fine-tuned the network to make it more suitable for our stain-free WBC dataset.

### 3.3. Training Process

Figure 2a shows the training process of the proposed network. Before standard training, we pre-trained the image enhancement network to obtain a better initial set of weight values. This is because optimized initial parameters can accelerate the convergence speed of gradient descent and are more likely to acquire models with low model error or low generalization error. The pre-training process is as follows: We expect the output image of the enhancement network to be approximately the same as the input image. Therefore, we set the input image and the corresponding label image as the same image. Then, during pre-training, fluctuations in the loss function value were observed, and training was stopped at the time when this value reached its minimum. The parameters trained at this point were saved as the initialization parameters.

After pre-training, we connected the output of the pre-trained enhancement network with the input of the transferred ResNet101 to achieve an end-to-end architecture. By fine-tuning our training dataset, the optimal weights of UR-Net were learned.

Moreover, to validate the effectiveness of our modified UR-Net model and pre-training, we trained three other models on the platform. The first model comprises solely a classification network, ResNet101. The second model is a combination of ResUNet and ResNet101, where ResUnet has not been modified. The third model is our proposed modified network, UR-Net. We, respectively, name them Net1, Net2, and Net3, and also name the pre-trained UR-Net Net4. These four models were trained on our WBC dataset, and the loss function of these four models are as follows:

(3) $$L=-\frac{1}{\left|X\right|}\sum_{i=0}^{\left|X\right|}\log(P(y^{i}\left|X^{i}\right.)),$$

where *X* represents the training samples, |*X*| represents the number of training samples, *i* represents the *i-*th sample, and *y* represents the ground truth. Other hyperparameter settings are shown in Table 1.

These four models were trained for 200 epochs and tested on the test dataset for each epoch, respectively and saved the weight parameters with the highest accuracy. These models were realized on a 64-bit Linux operating system with an NVIDIA RTX 3090 GPU based on the PyTorch framework and Python 3.9 version. To maximize the utilization of our hardware system, we set the batch size to 32 and the learning rate to 10^−4^.

## 4. Experimental Results and Discussion

Table 2 presents the results of different combinations of image enhancement network models and classification network models. For the image enhancement network models, we selected UNet and its enhanced versions, UNet++ and ResUNet. As for the image classification network models, we chose four commonly used models: VGG16, MobileNetV2, DenseNet121, and ResNet101. From the results, it can be observed that the combination of ResUNet and ResNet101 achieves the highest accuracy (81.78%). Although DenseNet121 (81.77%) performs comparably to ResNet101 in terms of accuracy, its deep convolutional layers result in long training times. Moreover, due to the relatively simple features of stain-free WBCs, excessively deep networks may fail to extract effective features. Therefore, we have selected ResUNet as the framework for our image enhancement network, and ResNet101 as the classification network to construct our model.

**Figure 3:**
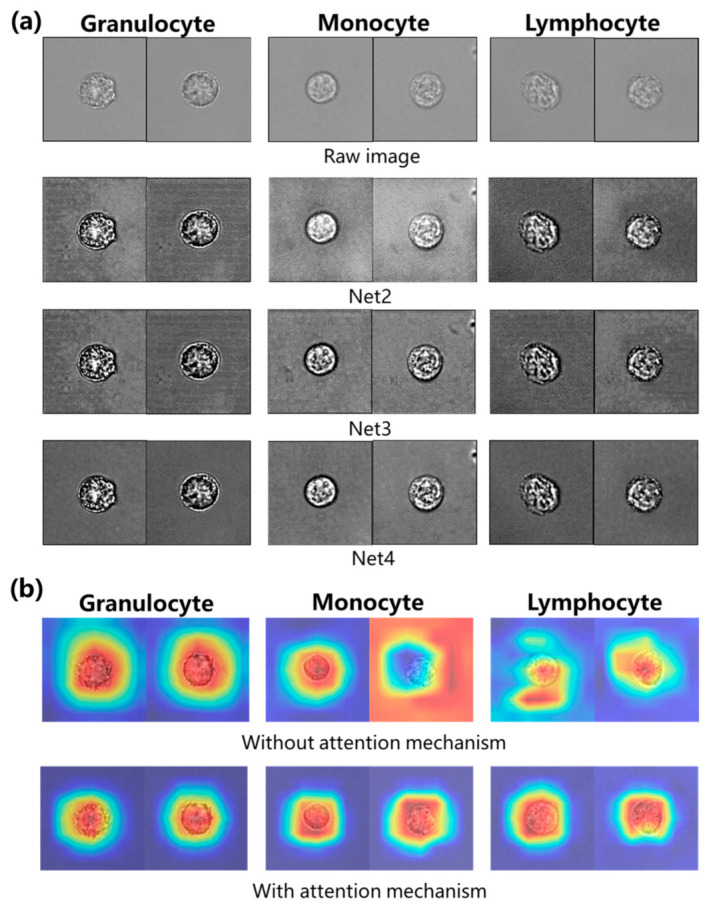
Experimental results. (**a**) Comparison of images before and after enhancement. (**b**) Comparison of thermal maps with and without attention mechanism. The color gradient ranging from blue to red represents gradually increasing weights.

Then, the four models mentioned above, namely Net1, Net2, Net3, and Net4, were trained for 200 epochs, and the optimal weight parameters were saved for each model. These weight parameters were employed to perform image enhancement on the test dataset, as shown in Figure 3a where the first row is the raw images of three types of WBCs, and the following three rows were the corresponding enhanced images generated by the model with enhanced networks, namely Net2, 3, and 4. Compared with the images before and after enhancement, the raw cell images appear blurry with insufficient details, whereas the contrast of the enhanced cell images is significantly improved, with more prominent light and dark areas and sharper edge details.

To gain a more intuitive understanding of the impact of channel attention mechanisms in network models, we utilized Grad CAM [43] to obtain thermal maps based on the weight of test dataset samples. As shown in Figure 3b, the color gradient ranging from blue to red represents gradually increasing weights, with higher weights indicating more salient cell features that the model should pay greater attention to. In this image, the first row features CAM images without attention mechanisms, where it is clear that the image background significantly interferes with the performance of the network model. In contrast, the second row with attention mechanisms shows that the classification network focuses more on the WBCs themselves rather than the background, which demonstrates the effectiveness of the channel attention mechanism.

**Figure 4:**
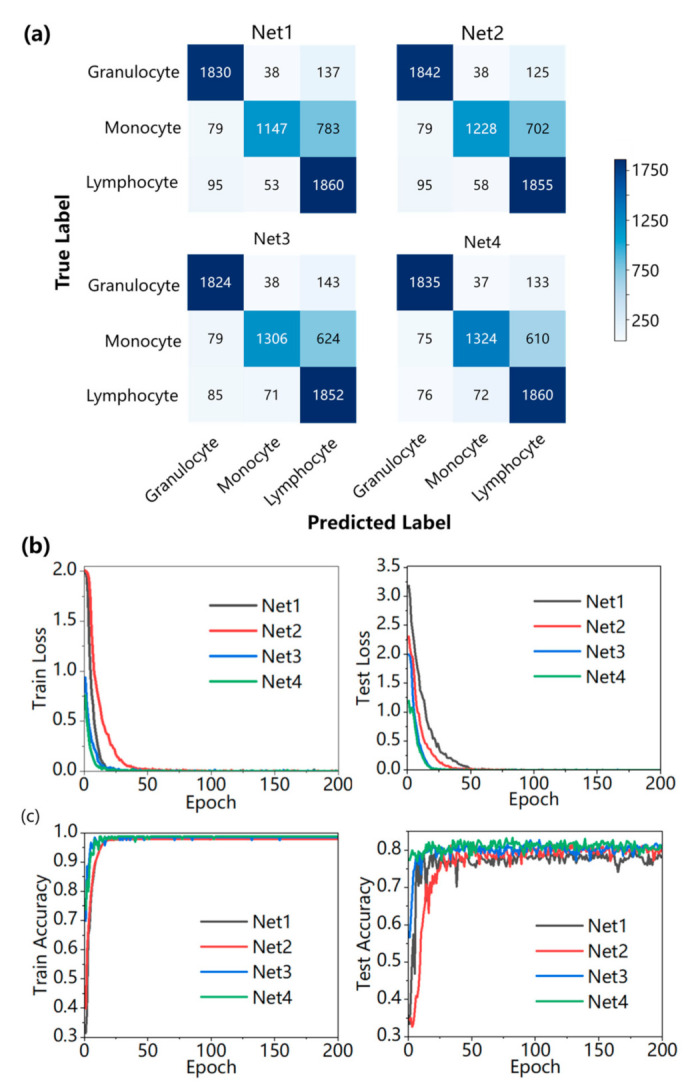
Experimental results. (**a**) Confusion matrix of four models. (**b**) Train loss and test loss of four models during training. (**c**) Train accuracy and test accuracy of four models during training.

The confusion matrix is a standard format for expressing accuracy evaluation, which can provide a more objective demonstration of the effectiveness and feasibility of our proposed model. Therefore, we employed the confusion matrix to evaluate our model. Figure 4a presents the confusion matrix for the four models at the highest test accuracy. From the confusion matrix, we calculated the recall, precision, accuracy, and F1 score to evaluate the performance of the three-class network models for stain-free WBCs as follows:

(4) $$\mathrm{Recall}=\frac{TP}{TP+FN},$$

(5) $$\mathrm{Precision}=\frac{TP}{TP+FP},$$

(6) $$\mathrm{Accuracy}=\frac{TP+TN}{TP+TN+FP+FN},$$

(7) $$F1\;\mathrm{score}=\frac{2\times \mathrm{Precision}\times \mathrm{Recall}}{\mathrm{Rrecision}+\mathrm{Recall}},$$

where *TP* and *TN* mean true positive and true negative, respectively, indicating that the prediction results are correct. *FP* and *FN* mean false positive and false negative, respectively, which represent errors in prediction results.

Table 3 shows the results of four metrics for four models, respectively. Overall, our proposed UR-Net model achieved the highest values in terms of F1 score (83.19%) and test accuracy (83.34%). In the comparison of test accuracy, it can be found that the models Net2, Net3, and Net4, which all have image enhancement networks, outperformed the single classification network Net1. Besides, our proposed modified model UR-Net indeed enhances the test accuracy by effectively extracting features and focusing more on WBCs themselves rather than other noise through the attention mechanism, thereby achieving higher results. However, from the perspective of recall metrics, the recall of monocytes among the four models is not high, and according to the confusion matrix, they are always classified as lymphocytes. The fundamental reason is that the number of monocytes in human blood during sampling is relatively small, resulting in an imbalanced initial dataset. Although dataset augmentation alleviates the imbalance to some extent, the true features of monocytes are still less represented compared to the other two types. Nevertheless, after pre-training, our model achieved nearly a 9% increase in recall for monocytes. Clearly, in the processed monocyte images by ResUNet, both shallow and deep features are more prominent, exhibiting higher performance in distinguishing them from lymphocytes.

Figure 4b,c show the training process of four models. Figure 4b displays the training loss values and test loss values of each model. From the figure of training loss, it can be observed that Net3 and Net4 converge faster than Net1 and Net2 which confirms that our modified model UR-Net can find the best direction for optimization during backpropagation. Figure 4c shows the training accuracy and testing accuracy of each model. The testing accuracy indicates that compared to Net1 without an enhanced network, the overall accuracy of the other three models is higher than Net1. In comparison to Net2 and Net3, Net2 exhibits significant fluctuation at the beginning of training, and Net3 achieves a higher overall accuracy. When comparing Net3 and Net4, it is observed that the pre-trained model has already achieved a high level of accuracy at the beginning, and converges more quickly. This is due to pre-training providing the CNN with an excellent initial value, enabling the network to converge towards the gradient descent direction more rapidly, thereby achieving higher accuracy in fewer training epochs. Compared with the training accuracy and testing accuracy, the training accuracy of the four models is around 98%. Although there is a certain gap between the training accuracy and the testing accuracy, as the training loss value decreases during the training process, the testing loss value continues to decrease and reaches convergence, and there is no decrease in testing accuracy. Therefore, these four models have not shown an overfitting phenomenon.

**Figure 5:**
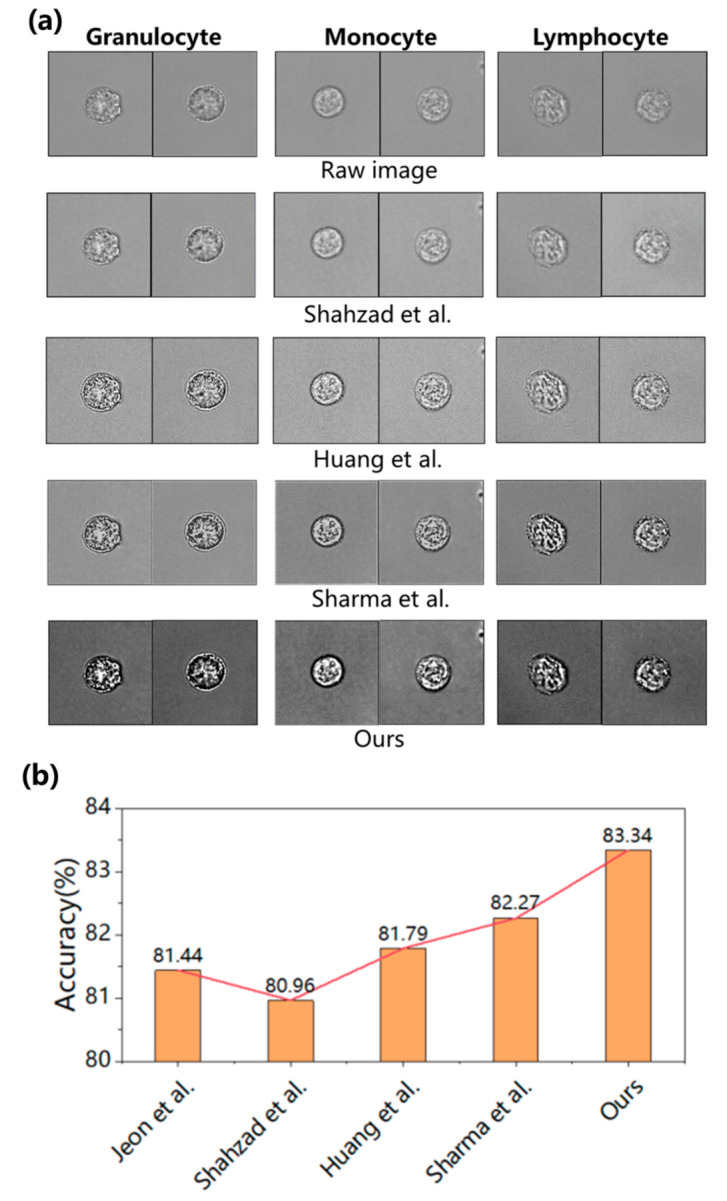
Experimental results. (**a**) Enhancement image of each work in our stain-free WBC dataset [23,25,32]. (**b**) Comparison of testing accuracy for each work [23,25,32,44].

In the end, we conducted a comparative analysis of our work with that of others. Jeon et al. [44] was our previous work, which did not use image enhancement methods. Shahzad et al. [25] used traditional image enhancement techniques while Huang et al. [32] used CNN for image enhancement. However, their image enhancement and classification were two independent modules. Sharma et al. [23] integrated a series of enhancement filters into the classification network. We reproduced the network models from these works and made slight modifications to adapt them to our WBC dataset, training them on our platform. Figure 5a presents the enhancement effect of each work, showing that the CNN-based method outperformed the traditional method in processing image details. Figure 5b presents the highest accuracy achieved during the training process, where we observed that traditional image enhancement techniques may not necessarily benefit subsequent image classification tasks. Conversely, integrating the enhancement technique into the classification network improved its classification performance. Notably, our proposed model exhibited better feature extraction capabilities, achieving the highest accuracy. At the same time, we calculated the recall, precision, F1 score, and accuracy of these models, as shown in Table 4. From the table, it can be concluded that the recall of monocytes has always been at a low level, indicating that the lack of initial dataset has a significant impact on the performance of network feature extraction.

The experimental results demonstrate that by integrating the ResUNet enhancement network with the ResNet101 classification network, the feature enhancement direction of the enhancement network is directed towards improving classification performance. Consequently, this integration enhances both the feature enhancement effectiveness and the classification accuracy simultaneously.

## 5. Conclusions

In this work, we proposed a unified architecture, UR-Net, for stain-free WBC classification. The architecture comprises an image enhancement network and a classification network, where the former is integrated into the latter to form an end-to-end model. The image enhancement network based on ResUNet utilizes upsampling, concatenation, residual structures, and attention mechanisms to enhance image features, and ResNet101 can fully extract and utilize features for accurate classification. Experimental results demonstrate that the proposed network model enhances images in a direction favorable for classification. Furthermore, an excellent initial value is learned by pre-training, which enables the model to converge at a faster speed and achieve higher accuracy with a limited number of training epochs. In comparison to the previous enhancement algorithms, the proposed model focuses on the identification performance, rather than observers’ perception. The results demonstrate that our proposed model achieves an optimal accuracy of 83.34%. However, there are limitations to this research. The proposed model has not been tested on WBC images collected in other adverse environments; therefore, the stability of the model remains to be investigated. In future work, we will continue to refine our experiments and optimize the network model to enhance the classification accuracy of stain-free WBCs. At the same time, the proposed method can also be applied to other high-level computer vision tasks like object detection.

## Figures and Tables

**Table 1:** Hyperparameter settings during pre-training and training process.

	Optimizer	Learning Rate	Momentum	Batch Size	Number of Epochs	Activation Function
Pre-train	SGD-M	10^−4^	0.9	32	100	LeakyReLU
Train	SGD-M	10^−4^	0.9	32	200	ReLU

**Table 2:** Accuracy of different combinations of enhancement networks and classification networks.

Model	Accuracy	Model	Accuracy	Model	Accuracy
UNet VGG16	78.01%	UNet++ VGG16	79.42%	ResUNet VGG16	79.46%
UNet MobileNetV2	78.14%	UNet++ MobileNetV2	79.32%	ResUNet MobileNetV2	79.28%
UNet DenseNet121	79.96%	UNet++ DenseNet121	81.62%	ResUNet DenseNet121	81.77%
UNetResNet101	80.60%	UNet++ ResNet101	81.43%	**ResUNet** **ResNet101**	**81.78%**

**Table 3:** Recall, precision, F1 score and test accuracy of four models with three types of WBCs.

Index	Types of WBC	Net1	Net2	Net3	Net4
Recall	Granulocyte	91.27%	**91.87%**	90.97%	91.52%
	Monocyte	57.09%	61.13%	65.01%	**65.90%**
	Lymphocyte	**92.63%**	92.38%	92.23%	**92.63%**
	Average	80.33%	81.79%	82.74%	**83.35%**
Precision	Granulocyte	91.32%	91.37%	91.75%	**92.40%**
	Monocyte	92.65%	**92.75%**	92.30%	92.39%
	Lymphocyte	66.91%	69.17%	70.71%	**71.46%**
	Average	83.63%	84.43%	84.92%	**85.42%**
F1 score	Granulocyte	91.29%	91.62%	91.36%	**91.96%**
	Monocyte	70.65%	73.69%	76.29%	**76.93%**
	Lymphocyte	77.70%	79.10%	80.05%	**80.68%**
	Average	79.88%	81.47%	82.57%	**83.19%**
Accuracy	Test accuracy	80.32%	81.78%	82.73%	**83.34%**

**Table 4:** Recall, precision, F1 score and test accuracy of each work.

Index	Types of WBC	Jeon et al. [44]	Shahzad et al. [25]	Huang et al. [32]	Sharma et al. [23]	Ours
Recall	Granulocyte	92.77%	93.57%	**93.77%**	91.72%	91.52%
	Monocyte	59.48%	56.10%	63.07%	64.56%	**65.90%**
	Lymphocyte	92.08%	**93.28%**	88.60%	90.54%	92.63%
	Average	81.44%	80.98%	81.81%	82.27%	**83.35%**
Precision	Granulocyte	91.99%	91.92%	91.44%	91.13%	**92.40%**
	Monocyte	90.05%	91.03%	88.54%	88.90%	**92.39%**
	Lymphocyte	69.17%	68.28%	70.18%	71.43%	**71.46%**
	Average	83.74%	83.74%	83.39%	83.82%	**85.42%**
F1 score	Granulocyte	92.38%	**92.73%**	92.59%	91.42%	91.96%
	Monocyte	71.64%	69.42%	73.66%	74.80%	**76.93%**
	Lymphocyte	79.00%	78.85%	78.32%	79.86%	**80.68%**
	Average	81.01%	80.33%	81.52%	82.03%	**83.19%**
Accuracy	Test accuracy	81.44%	80.96%	81.79%	82.27%	**83.34%**

## Data Availability

Data available on request due to privacy restrictions.
